# The effects of plasma chromium on lipid profile, glucose metabolism and cardiovascular risk in type 2 diabetes mellitus. A case - control study

**DOI:** 10.1371/journal.pone.0197977

**Published:** 2018-07-05

**Authors:** Robert Amadu Ngala, Martin Akilla Awe, Paul Nsiah

**Affiliations:** 1 Department of Molecular Medicine, School of Medical Science, Kwame Nkrumah University of Science and Technology (KNUST), Kumasi, Ghana; 2 Department of Chemical Pathology, University of Cape Coast,- Ghana; Universita degli Studi Magna Graecia di Catanzaro Scuola di Medicina e Chirurgia, ITALY

## Abstract

**Background:**

The study was aimed at determining the effect of plasma chromium concentration on the metabolism of glucose, and lipids and their subsequent cardiovascular risk in patients with type 2 diabetes in the Bolgatanga district of Ghana.

**Material and methods:**

Fasting blood glucose and lipids profile were determined by enzymatic assay using the BT 5000^®^ Random Access Chemistry Analyzer. Fasting serum insulin and High sensitive C-reactive protein were determined by ELISA, a solid phase direct sandwich immunoassay method. HOMA-IR, which is based on fasting blood sample for insulin and glucose concentrations measured in a single blood sample, was used to calculate insulin resistance. Plasma chromium was measured using an atomic Absorption Spectrometer.

**Results:**

Patientswith diabeteshad significantly (p<0.0001) increased LDL, TC, TG, VLDL, insulin, CRP and HOMAIR and a significantly reduced plasma chromium (p<0.0001) (0.53± 0.02μg/l and 0.11±0.01μg/l control and case respectively). Low Cr (p ≤0.001) was associated with high blood pressure, obesity and lipid dysregulation. Plasma Cr significantly correlated negatively with blood pressure and LDL.

**Conclusion:**

Lower plasma Cr level was associated with hyperglycaemia, hyperinsulinemia, hypertension, insulin resistance and high inflammation marker HsCRP.

## Introduction

Excessive ingestion of high fat and carbohydrate diets are the major macronutrients that have been associated with the development of diabetes and obesity. However, micronutrients, including trace elements particularly chromium (Cr), are also known to play significant roles in lipid and carbohydrate metabolism [1–3] and thereby contributing to the etiology of diabetes, obesity and cardiovascular diseases (CVD)[4–6] Chromium is a transition metal and trivalent chromium has been referred to as the ‘glucose tolerance factor’ and implicated in the regulation of glucose and lipid metabolism[7]. The exact mechanism of action remains unclear; however, several mechanisms have been proposed to explain the signaling process. Chromium acts as a cofactor or secondary messenger to insulin, improves insulin sensitivity and facilitate glucose utilization by insulin target tissues and[8–9]. Chromium also improves insulin affinity to its receptors, and also activates insulin receptor kinases and at the same time inhibiting insulin receptor phosphatases [10]. Chromodulin, the Cr-binding protein, promotes tyrosine kinase activity of insulin receptor during response to insulin [11]. This hypothesis is supported by the observation that Cr deficiency leads to elevated blood glucose, total cholesterol and triglycerides and decreased in high density lipoproteins (HDL) and insulin sensitivity even in humans on normal diets [12, 13]. Epidemiological evidence for the incidence of Cr deficiency is limited; however, several studies in humans and animals experiments support the beneficial effects of Cr supplementation on glucose metabolism and insulin sensitivity [14, 15]. The relationship between chromium and insulin resistance has been further demonstrated in patients with high insulin resistance, having a corresponding high Cr excretion in urine in comparison to those patients with diabetes who have a lesser degree of insulin resistance [16]. However, some studies reported minimal beneficial effects of Cr [17, 18] in patients with diabetes.

This study attempts to evaluate the correlations between plasma Cr levels, insulin resistance, glucose utilization, dyslipidemia and cardiovascular risk in type 2 diabetics attending the Bolgatanga Regional Hospital- Ghana.

## Materials and methods

### Subjects

A case—control study was carried out at the outpatient department (OPD) and theDiabetes Clinic in the Upper East Regional Hospital (Bolgatanga-Ghana), among subjects visiting the facility as patients with diabetesor qualified blood donors, between the period August 2014 to July 2015. The study population was made up of 163 enrolled diagnosedpatients with type 2 diabetes, consisting of 68 males and 95 femalesaged between 35–65 years who reported at the diabetes clinic and 168consisting 70 males and 98 females of healthy non-diabetic volunteers from the same locality aged matched with the patients with diabeteswere used as the control. A total of 331 subjects were recruited for this study.Patients with diabeteswhose life style had not changed, pertaining to exercise and dietary habit in the past four weeks were enrolled, otherwise excluded from the study,also excluded from the study were pregnant women. This information was obtained by simple interview through a designed questionnaire.

### Ethical clearance approval

All procedures were approved by the Committee on Human Research Publication and Ethics of School of Medical Sciences, KNUST Kumasi, Ghana (CHRPE/Student/113/09) and the committee on human research publication and ethic of the Navrongo Health Research Centre. A written consent form was completed and signed/thumb-printed by all the participants who were recruited into the study after the study was explained to them in a language they understand.

### Anthropometric parameter measurements

Body weight was measured (to the nearest 0.5 kilogram). Height was measured (to the nearest 1.0 millimeter), Waist circumference (WC), Hip circumference (HC) and blood pressure were measured as recommended by the World Health Organization (WHO, 2006, WHO, 1998).

### Sample collection

About 10.0 ml of venous blood samples from overnight fasting subjects was aseptically collected from the median antecubital or cephalic veins. 4.0ml of the blood was then dispensed into labelled plain BD vacutainer® tubes for the lipid profile and other biochemical parameters measurements and. 1.0 ml in to fluoride oxalate coated tubes (Becton Dickenson, Plymouth, UK) for fasting blood glucose determination. 5.0 ml of blood was also dispensed into another labelled trace metal free evacuated tube containing EDTA (BD, Plymouth, UK, Royal blue top Vacutainer ®) for Cr assay. Samples for blood glucose assay were immediately analysed. After clotting, blood sample in the plain tubes were centrifuged and the serum stored at -20°C until ready for analysis for chromium (Cr), lipid profile and other biochemical parameters.

### Biochemistry analysis

Lipid profile and blood glucose were measured using enzymatic methods, whilst insulin and HsCRP were assayed using ELISA method at the Chemical Pathology laboratory at the Kwame Nkrumah University of Science and Technology, Kumasi, Ghana and **HOMA-IR** calculated.

Chromium was measured with an atomic Absorption Spectrometer (VARIAN AA 240FS- Atomic Absorption Spectrometer) at the Atomic Energy, Chemistry laboratory, Accra,Ghana.

### Statistical analysis

All statistical analyses were performed using GraphPad prism 5.0 (Graph PadSoftware, San Diego California USA, www.graphpad.com) and Microsoft Excel 2007. Continuous variables were expressed as mean ± SEM, while categorical variables expressed as proportion. Comparisons of the subjects and control group, were performed using unpaired t tests, χ2 tests, or Fisher exact tests, where appropriate. One-sample t-test was used in comparison between means Cr levels within group variable. Odds ratio and their 95% confidence intervals were used to quantify the risk of subjects in highly risk population. A level of p<0.05 was acceptable as statistically significant unless otherwise stated. Comparison of clinical variables, biochemical markers and anthropometrics between diabetics and control groups was by Pearson Correlation Coefficient. Correlation was significant at the 0.05, 0.01, 0.001 levels (2-tailed)

## Results

Table 1 above depicts a case-control study with a higher proportion of the participants being females (58.3%) as compared to males (41.7%). The total participants enrolled into the study were 331, of which diabetics were 163 and 168 non-diabetics (control group). There was no significant difference in age between the case and control groups (52.43 ± 0.69 vs. 51.96 ± 1.23, p = 0.7232). In the case group, subjects had a significant increase in blood pressure (SBP and DBP) compared to the controls group (p<0.0001). Patients with diabeteshad higher values in all of the anthropometric measurements; weight, BMI, HC, WC, WHR, WHtR, WTR and mid upper arm circumference as compared to non-diabetics (control group).

**Table 1:** General demographic, clinical and anthropometric characteristics of study participants.

Variables	Total (n = 331)	Control (n = 168)	Case (n = 163)	p-value
AGE (years)	52.19 ± 0.72	51.96 ± 1.23	52.43 ± 0.69	0.7232
**Gender**				
Male	138 (41.7%)	78 (46.4%)	60 (36.8%)	0.0943
Female	193 (58.3%)	90 (53.6%)	103 (63.2%)	0.0943
**Blood pressure**				
SBP (mmHg)	126.67 ± 1.27	120.5 ± 1.29	138.7 ± 1.67	< 0.0001
DBP (mmHg)	81.67 ± 0.82	76.53 ± 0.88	89.20 ± 1.02	< 0.0001
**Anthropometrics**				
Height (m)	1.65 ± 0.01	1.67 ± 0.02	1.64 ± 0.01	< 0.0001
Weight (kg)	68.11 ± 0.76	62.71 ± 1.05	73.50 ± 1.13	< 0.0001
BMI (kg/m^2^)	26.05 ± 0.28	23.57 ± 0.38	27.52 ± 0.41	< 0.0001
WC (cm)	85.92 ± 0.78	79.95 ± 0.86	92.76 ± 0.96	< 0.0001
HC (cm)	95.88 ± 0.65	91.32 ± 0.78	98.92 ± 0.88	< 0.0001
WHR	0.89 ± 0.01	0.88 ± 0.01	0.94 ± 0.01	< 0.0001
WHtR	0.52 ± 0.01	0.49± 0.01	0.57 ± 0.01	< 0.0001
THIGHT (cm)	49.51 ± 0.42	46.21 ± 0.60	50.25 ± 0.54	< 0.0001
WTR	1.75 ± 0.01	1.75 ± 0.02	1.86 ± 0.02	< 0.0001
MUAC (cm)	28.65 ± 0.24	26.77 ± 0.29	29.72 ± 0.36	< 0.0001

Comparison of the general demographic, clinical and anthropometric characteristics of study participants

Comparison between means was done using un-paired t-test. p < 0.05 was considered statistically significant. WHR: Waist to hip ratio; WTR: Waist to thigh ratio; WHtR: Waist to height ratio; BMI: Body mass index; WC: Waist circumference; HC: Hip circumference; MUAC: Mid upper arm circumference.

The diabetic group had significantly (p<0.0001) increased LDL, TC, TG, VLDL. HDL level did not show any statistically significantly change (p = 0.3420). Mean FBG level was about two times higher in patients with diabetes (p< 0.0001). The mean plasma insulin, HsCRP and HOMAIR were also significantly higher in diabetic group than in the control group (p<0.05) but about four times lower level of Cr (p<0.05) (Table 2).

**Table 2:** Biochemical characteristics of the study participants.

Variables	Total (n = 331)	Control (n = 168)	Case (n = 163)	p-value
**Lipid profile**				
TC (mmol/l)	4.04 ± 0.11	3.74 ± 0.15	4.59 ± 0.10	< 0.0001
TG (mmol/l)	1.27 ± 0.05	1.05 ± 0.05	1.51 ± 0.07	< 0.0001
HDL-C (mmol/l)	1.38 ± 0.05	1.38 ± 0.07	1.46 ± 0.05	0.3420
LDL-C (mmol/l)	3.22 ± 0.11	2.13 ± 0.12	4.19 ± 0.09	< 0.0001
VLDL-c (mmol/l)	0.59 ± 0.02	0.48 ± 0.02	0.69 ± 0.03	< 0.0001
TC/ HDL	2.93 ± 0.13	2.71± 0.16	3.18± 0.11	0.3220
**FBG (mmol/l)**	7.39 ± 0.25	4.56 ± 0.08	10.22 ± 0.34	< 0.0001
**Biomarkers**				
Cr (μg/l)	0.32 ± 0.01	0.56 ± 0.02	0.11 ± 0.01	< 0.0001
Insulin (μIU/mL)	9.44 ± 0.68	6.97 ± 0.35	11.77 ± 1.20	0.0001
HsCRP(mg/L)	7.39 ± 0.32	6.36 ± 0.41	7.76 ± 0.47	0.0229
HOMA-IR	3.59 ± 0.37	1.41 ± 0.07	5.48 ± 0.65	< 0.0001

The general biochemical parameters of the study population, depicting atherogenic lipid profile tendency in the diabetic (Case group) compared to the non-diabetics (control group). Comparison between means was done using un-paired t-test. p<0.05 was considered statistically significant.

FBG: Fasting blood Glucose; hsCRP: high sensitive C-reactive protein; HOMA-IR: Homeostasis model assessment of insulin resistance; Cr: chromium; TC; total cholesterol; TG: triglyceride; HDL-C:high density lipoprotein cholesterol; LDL-C:low density lipoprotein cholesterol; VLDL-C:very low density lipoprotein cholesterol.

Obesity, (measurement defined by BMI ≥30Kg/M^2^, WC (male ≥ 102 cm and female≥ 88 cm)WHR (men ≥ 1.0 and women and ≥ 0.85)WHtR(≥0.5), WTR (≥1.95) was significantly associated with lower Cr level than the non-obese (p<0.05). Plasma Cr was markedly decreased in patients with diabetes compared to normoglycemic control group (p<0.0001). However, Cr level was not statistically significantly changed between the dyslipidaemic and the control (p>0.05) when using both the WHO and NCEP III criteria for defining metabolic syndrome (Table 3).

**Table 3:** Comparison between plasma Chromium levels in obesity, diabetes and dyslipidaemia in the study population.

Parameters	Total (n = 331) n (%)	Cr level (μg/l) Mean ± SEM	p-value
**WC (cm)**			
central obesity	108 (33.0%)	0.133 ± 0.01	<0.0001
Normal	223 (67.0%)	0.308 ± 0.02	
**BMI (Kg/m**^**2**^**)**			
Normal	220 (66.5%)	0.319 ± 0.03	1(reference)
Overweight	61 (18.4%)	0.217 ± 0.01	0.036
Obese	50 (15.1%)	0.139 ± 0.02	0.003
**WHR**			
Normal	141 (42.6%)	0.355 ± 0.03	<0.0001
Obese	190 (57.4%)	0.138 ± 0.01	
**WHtR**			
Normal	147 (44.4%)	0.358 ± 0.02	<0.0001
Obese	184 (56.6%)	0.132 ± 0.01	
**WTR**			
Normal	268 (81.8%)	0.281 ± 0.01	<0.0001
Obese	63 (18.2%)	0.092 ± 0.02	
**FBG**			
Normal	181 (54.7%)	0.397 ± 0.02	<0.0001
Diabetic	150 (45.3%)	0.078 ± 0.01	
**TG (WHO Criteria)**			
Normal	268 (81.0%)	0.263 ± 0.01	0.844
Dyslipidaemia	63 (19.0%)	0.220 ± 0.04	
**TG (NCEP III Criteria)**			
Normal	259 (78.2%)	0.256 ± 0.01	0.828
Dyslipidaemia	72 (21.8%)	0.230 ± 0.04	

Comparison of plasma Cr level in obesity, diabetes and dyslipidaemia in the study population

n (%): frequency (percentage). One-sample t-test was used in comparison between means Cr levels.p < 0.05 was considered statistically significant

BG: Fasting blood Glucose, WHR: Waist to hip ratio; WTR: Waist to thigh ratio; WHtR: Waist to height ratio; BMI: Body mass index; WC: Waist circumference; HC: Hip circumference TG; triglycerides

Patients with diabetesand significantly low plasma Cr level(p≤0.001) had increased blood pressures (SBP and DBP) and had a tendency to obesity as indicated by high values of their anthropometric measurements (BMI = 27.8 ± 0.53, WHR = 0.95 ± 0.01, WHtR, = 0.58 ± 0.01 and WTR = 1.89 ± 0.01). Also plasma lipid levels (TC, TG, and LDL-C), fasting blood sugar levels, hyperinsulinemia (insulin levels) and high insulin resistant (high HOMA-IR values) were significantly higher in subjects who had significantly low plasma Cr level (p ≤ 0.001). However, the mean level of HDL-cholesterol was increased in those with high plasma Cr level though not statistically significant (p>0.05) (Table 4).

**Table 4:** The effects of plasma chromium concentration on anthropometry, lipid profile and insulin sensitivity in the diabetics.

Variables	Cr ≤ 0.001 (n = 126)	Cr > 0.001 (n = 205)	p-value
**Blood pressure**			
SBP (mmHg)	144.2 ± 2.17	120.4 ± 1.36	<0.0001
DBP (mmHg)	91.3 ± 1.26	79.3 ± 0.92	<0.0001
**Anthropometrics**			
BMI (Kg/m2)	27.8 ± 0.53	25.3 ± 0.32	0.0078
WHR	0.95 ± 0.01	0.85 ± 0.01	<0.0001
WHtR	0.58 ± 0.01	0.53 ± 0.01	<0.0001
WTR	1.89 ± 0.01	1.68 ± 0.01	<0.0001
**Lipid profile**			
TC (mmol/l)	4.57 ± 0.13	3.69 ± 0.14	0.001
TG (mmol/l)	1.55 ± 0.10	1.13 ± 0.01	0.0023
HDL-C (mmol/l)	1.39 ± 0.06	1.45 ± 0.06	0.569
LDL-C (mmol/l)	4.37 ± 0.12	2.98 ± 0.13	<0.0001
**FBG (mmol/l)**	10.37 ± 0.45	6.44 ± 0.24	<0.0001
**Biomarkers**			
Insulin (μIU/mL)	11.51 ± 1.48	8.46 ± 0.72	0.0364
HsCRP (mg/L)	7.88 ± 0.61	7.58 ± 0.36	0.556
HOMA-IR	5.81 ± 0.96	3.13 ± 0.26	<0.0001

Comparison between means was done using un-paired t-test. p<0.05 was considered statistically significant

FBG: Fasting blood Glucose; hsCRP: high sensitive C-reactive protein; HOMA-IR: Homeostasis model assessment of insulin resistance; Cr: chromium; TC; total cholesterol; TG: triglyceride; HDL-C:high density lipoprotein cholesterol; LDL-C:low density lipoprotein cholesterol; VLDL-C:very low density lipoprotein cholesterol, WHR: Waist to hip ratio; WHtR: BMI: Body mass index

There was no significant correlation between the lipids profiles and Cr of the patients with diabetes. However, plasma Cr was significantly but negative correlated with systolic and diastolic blood pressure and LDL (r = - 0.200, r = -0.189 r = -0.180) respectively among the controls. HDL was positively correlated with TC in the diabetics (r = 0.324) and HOMA-IR in the controls (r = 0.210), while insulin was significantly negatively correlated with hsCRP (r = -0.214) and positively correlated with HOMA-IR, (r = 0.942) in the controls (Table 5).

**Table 5:** Pearson Correlation between Clinical Variables and biochemical markers for cases (Upper Right-Hand Side) and Control (Lower Left-Hand Side).

	Cr	SBP	DBP	TC	HDL	TG	LDL	insulin	hsCRP	HOMAIR
Cr		0.059	0.047	0.016	0.060	0.073	0.004	-0.072	0.053	-0.086
SBP	-0.200*		0.663**	0.206**	0.027	0.079	0.198*	-0.108	-0.092	-0.172*
DBP	-0.189*	0.686**		0.138	0.089	0.048	0.149	-0.163*	-0.161*	-0.222**
TC	-0.126	0.014	0.107		0.324**	0.372**	0.965**	-0.050	0.028	-0.054
HDL	0.117	-0.018	0.051	0.431**		-0.121	0.237**	-0.020	-0.027	-0.004
TG	0.132	0.167*	0.176*	0.117	0.013		0.258**	-0.083	0.134	-0.044
LDL	-0.180*	-0.003	0.095	0.939**	0.185*	0.120		-0.041	0.006	-0.049
insulin	-0.054	0.035	**0.031**	-0.057	0.151	-0.119	-0.077		0.084	0.845**
hsCRP	-0.045	0.077	0.203*	0.046	-0.088	0.197*	0.084	-0.214*		0.170*
HOMAIR	0.000	0.060	0.087	-0.067	0.210*	-0.032	-0.102	0.942**	-0.192*	

Pearson Correlation between Clinical Variables and biochemical markers for cases (Upperht-Hand Side) and controls (Lower Left-Hand Side) are indicated in Table 5

Values represent correlation coefficient (r).

* Correlation is significant at P < 0.05 level (2-tailed)

** Correlation is significant at P < 0.01level (2 –tailed)

*** Correlation is significant at P <0.001 level (2 –tailed). Underline and boldface represent correlation coefficient (0.3 < r < 0.5). Cr: Chromium; SBP: Systolic blood pressure; DBP: diastolic blood pressure; TC: Total Cholesterol; TG: Triglyceride; LDL: Low density lipoprotein cholesterol; HSCRP: Highly sensitive c -reactive protein; HOMA-IR: Homeostasis model assessment of insulin resistance; HDL-c; high density lipoprotein cholesterol

## Discussion

The metabolism of chromium is affected by several factors including, stress, diet, exercise, and diabetes[19] and increased intake of simple sugars results in increased chromium loss [20–22]_._ Therefore, subjects whose life style had not changed, pertaining to exercise and dietary habit in the past four weeks were enrolled for the study. The ability to covert chromium into a more useful form may be the key difference between chromium metabolisms in the diabetics compared to the non-diabetes patients. Patients with diabetes have been shown to have higher chromium absorption but also greater chromium excretion [23]. Individuals with diabetes tend to lose the ability to convert inorganic chromium to a useable form [24,25]. Perhaps, similar to the observation that diabetic mice lose the ability to convert inorganic chromium to a useable organic form (organic chromium) that activates insulin [22]

Plasma lipids except HDL were significantly elevated in the patients with diabetes (Table 2). Lipoprotein abnormalities are usually present in type 2 diabetes, which includes hypertriglyceridemia, increased LDL and reduced plasma HDL- cholesterol and also LDL’s are converted to smaller, and more atherogenic, lipoproteins [26,27]. These abnormalities are linked to the increased metabolism of apolipoprotein B (apoB). Experimental evidence suggests that regulation of apoB production, increase lipolysis in adipocytes due to poor insulin activity results in increased fatty acid release from adipose cells. Insulin has also been shown to directly increase the degradation of apoB which ameliorates dyslipidaemia [28–29]. Therefore, insulin deficiency or hepatic insulin resistance may increase the secretion of apoB, and upregulate VLDL and LDL and increase cardiovascular risk. In this study even though plasma lipids were elevated, they were still within the physiological ranges and the atherogenic or cardiovascular risk (total cholesterol / HDL) was low. The significantly low plasma chromium in patients with type 2 diabetes agrees with the derailed lipid metabolism. The low cardiovascular risk may be attributed to the life style of the subjects. Increased exercise or energy expenditure has been shown to improve lipid profile [30]. Indeed many of the subjects are subsistent farmers or petty traders and these types of occupations involves a lot of movement hence there is very minimal sedentary life style in this region compared to the other urban areas in the country. The staple diets in this population are rich in vegetable fibre. However, apart from the lipid profile, the metabolism of the biomarkers which are affected by chromium metabolism may be predictors of cardiovascular risk. CRP, insulin and HOMAIR were all significantly elevated (Table 2) in patients with diabetes. CRP significantly correlated positively with diastolic blood pressure, triglycerides and HOMAIR and negatively correlated with insulin (Table 5). CRP is an acute phase reactant and a member of the pentraxin family of innate response proteins [31]. Baseline levels of CRP are predictive of risk of myocardial infarction, and stroke. Evidence suggests that CRP may be directly linked to atherogenesis, and that arterial plaque can produce CRP, independent of the hepatic pathways [32]. Recent data suggest that CRP may play a direct role in atherogenesis by inducing the expression of intercellular adhesion molecule-1 (ICAM-1) and vascular adhesion molecule-1 (VCAM-1) by endothelial cells [33,34] and mediates the monocyte chemoattractant protein-1(MCP-1) induction. CRP activates complement [35], as well as mediating the uptake of LDL by macrophages [36]. The plasma level of the CRP of both the control and patients with diabetes though was more than twice the value (6.36±0.41 and 7.76±0.47 mg/l respectively) reported by the American Heart foundation as cardiovascular risk (greater than 3.0mg/l) [37], this may not necessarily imply the subjects have increased cardiovascular risk. These high values may be due to an immune response. Indeed it has been shown that in the Garu-Tempani district in the Upper East region of Ghana, CRP values as high as 24.6mg/l were recorded and this was attributed to immune response to *P*. *Falciparum*parasitaemia[38]. This work is sited around similar setting in the Upper East region and therefore the high CRP is not surprising because samples were collected throughout the year during some point when malaria or *P*. *Falciparum parasitaemia* is high. Besides, it has also been shown that CRP levels abnormalities are changed by the ethnicity factor [39], possibly also accounting for the high level in this work. Since CRP in the diabetes patients was significantly higher than in the control, the results of CRP, triglycerides and HOMAIR may therefore be predictive of an increased cardiovascular risk independent of the lipid profile.

Plasma insulin has been shown to be predictive of cardiovascular risk. High insulin levels may constitute a more sensitive predictor of CHD than the degree of glucose intolerance, it may be useful to avoid excessive plasma insulin concentration, and even to lower its level [35]

The metabolism of CRP and insulin seems to be regulated by the plasma levels of chromium (Table 4). When Cr levels are significantly lower (diabetics) insulin is upregulated (***coefficient (r);***-0.072) whereas when plasma levels of Cr are significantly higher (in control subjects) insulin and CRP levels are downregulated (***coefficient (r);***-0.054 and -0.045 respectively). The mechanisms by which Cr metabolism affects plasma insulin and CRP levels are unclear, even though some studies have shown it improves insulin sensitivity [17,18,40,41]. In this study, it is suggested, it may improve insulin and CRP secretion. Similarly, significantly decreased plasma Cr (p ≤0.001) concentration was associated with significantly increased plasma total cholesterol, LDL and VLDL. Low Cr levels were also associated with both an increased systolic and diastolic pressure (p<0.0001). Indeed, normal Cr levels in the control subjects, were significantly correlated negatively both with systolic and diastolic blood pressure whereas low Cr level was contrary indicated in patients with diabetes as well (Table 5). Hence, high plasma Cr seems to ameliorate hypertension. Cr also seems to play a role in the anthropometry [42] of the subjects (Table 3). High plasma Cr was associated with fat distribution; WHtR, WC and WHR were significantly lower at significantly greater (p> 0.001) plasma Cr levels (Table 3 and Table 4). However, Cr effect was not significant on dyslipidaemia as determined by the WHO and NCEP III Criteria (Table 3)

## Conclusion

Lower plasma Cr level was associated with hyperglycaemia, hyperinsulinemia, hypertension, insulin resistance and CRP. High plasma Cr was also associated with favourable fat distribution: WHtR, WC and WHR were significantly lower at high plasma Cr levels. Plasma chromium concentration therefore ameliorate glucose and lipid metabolism. High plasma chromium there reduces cardiovascular risk.

### Limitation

Plasma chromium levels are also affected by several factors including, stress, diet, exercise. It was not possible to evaluate these confounding factors on chromium levels in the patients with diabetes.

## Supporting information

S1 File (XLSX)

S2 File (DOCX)

## Abbreviations

HOMA-IR: Homeostasis Model Assessment Index-Insulin Resistance
HsCRP: High-Sensitivity C—reactive protein
BMI: Body Mass Index
