# Supplementary material for: The effects of plasma chromium on lipid profile, glucose metabolism and cardiovascular risk in type 2 diabetes mellitus. A case - control study
Source: PLoS One. 2018 Jul 5;13(7):e0197977. doi: 10.1371/journal.pone.0197977 (PMC6033385; doi:10.1371/journal.pone.0197977)
Supplement: S2 File — (DOCX) [file pone.0197977.s002.docx]

Methodology

Subjects

A case – control study was carried out at the outpatient department (OPD) and the Diabetic Clinic of the Upper East Regional Hospital (Bolgatanga-Ghana), among diabetic subjects visiting the facility and qualified blood donors as control subjects. The study population was made up of 161 enrolled diagnosed type 2 diabetics aged between 35-65 years who reported at the diabetic clinic and 165 healthy non-diabetic volunteers from the same locality aged matched with the diabetics were used as the control.

Diabetes subjects whose life style had not changed, pertaining to exercise and dietary habit in the past four weeks were enrolled for the study. This information was obtained by simple interview through a designed questionnaire.

Ethical

All procedures were approved by the Committee on Human Research Publication and Ethics of School of Medical Sciences, KNUST Kumasi, Ghana (CHRPE/Student/113/09) and the committee on human research publication and ethic of the Navrongo Health Research Centre. A written consent form was completed and signed/thumb-printed by all the participants who were recruited into the study after the study was explained to them in a language they understand.

Anthropometric parameter measurements

Body weight was measured (to the nearest 0.5 kilogram) with the subject standing on an electrical weighing scale wearing light clothing. The weighing scale was calibrated using known weights from Ghana standard authority. Height was measured (to the nearest 1.0 millimeter) with the Subject standing in an erect position against a vertical scale of portable stadiometer, with their heads positioned so that the top of the external auditory meatus was in level with the inferior margin of the bony orbit. Using tape measure, Waist circumference (WC) was measured midway between the lower rib margin and the iliac crest at the end of normal expiration. Hip circumference (HC) was measured on the maximal circumference over the buttocks and at the level of greater trochanters. The measurements of the thighs were taken in the mid-way between the inguinal fold and the proximal border of the patella. All measurements were made in duplicates, to the nearest centimetre and the mean values were used for subsequent analysis, as recommended by the World Health Organization (WHO, 2006). BMI was calculated as weight in kilograms divided by squared of the height in meter, waist- to- hip ratio were calculated as WC divided by HC and the waist- to- height ratio was calculated as WC divided by Ht in meters.

##

Blood pressures were measured two times in a seated position after 15 min of rest using a standard mercury sphygmomanometer (by observing the appearance of the first and the disappearance of the fifth Korotkoff sound); measurements were made between the hours of 7:00am and 10:00 am [1] **.** High systolic blood pressure (SBP) and high diastolic blood pressure (DBP) were defined using WHO, 1998 criteria.

Sample collection**:**

About 10.0 ml of venous blood samples from overnight fasting subjects was aseptically collected from the median antecubital or cephalic veins. 4.0ml of the blood was then dispensed into labelled plain BD vacutainer® tubes for the lipid profile and other biochemical parameters and. 1.0 ml in to fluoride oxalate coated tubes (Becton Dickenson, Plymouth, UK) for fasting blood glucose determination. 5.0 ml of blood was also dispensed into another labelled trace metal free evacuated tube containing EDTA (BD, Plymouth, UK. Royal blue top Vacutainer®), for Cr assay. Samples for blood glucose assay were immediately analysed. After clotting, blood sample in the plain tubes were centrifuged and the serum stored at -20°C until ready for analysis for chromium (Cr), and the lipid profile and other biochemical parameters

Biochemistry analysis

Lipid profile, blood glucose, insulin and HsCRP were assayed at the Chemical Pathology laboratory at the Kwame Nkrumah University of Science and Technology, Kumasi, Ghana, whilst plasma chromium was determined at the Atomic Energy, Chemistry laboratory, Accra, Ghana.

Serum fasting insulin

Serum insulin measurement kit was obtained from CALBIOTECH, through a local agent. Fasting serum insulin was determined by using ELISA method; a solid phase direct sandwich immunoassay method, with precision coefficient of variation of both intra-Assay and inter-Assay on two serum samples as 6.3% ,8.1% and 8.5%, 7.4% respectively**.** The optical density (OD) was measured by spectrophotometer (MULTISKAN EX, Thermo Electron Corporation). During incubation, insulin in the sample reacts with enzyme Horse radish peroxidase (HRP)-conjugated anti-insulin antibody and anti-insulin antibody bound to micro-titration well. Washing removed unbound enzyme labelled antibody. The bound HRP complex was detected by reaction with tetramethylbenzidine substrate (TMB). The reaction was stopped by adding 50µl of 0.5M sulphuric acid (stopping solution) to give a colorimetric endpoint that was read using ELISA reader at 450 nm.

HsCRP

High sensitive C-reactive protein (HsCRP) was determined using ELISA method. The reagent kit from CALBIOTECH, based on a solid phase direct sandwich method with precision coefficient of variation of intra-Assay and inter-Assay on three serum samples as 5.05%, 5.28%, 9.59% and 8.51%, 8.34%, 7.95% respectively was used. Six standards each for high sensitivity CRP were provided by the manufacturer and used to draw the standard curve from which the various absorbance tests concentration was interpolated. The samples and anti-CRP-HRP conjugate were added to the wells coated with MAb to CRP. CRP in the patient’s serum binds to anti-CRP MAb on the well and the anti-CRP second antibody then binds to CRP. Unbound protein and HRP conjugate was washed off with wash buffer. The reaction was stopped by the addition of 50µl sulphuric acid (stopping solution) to give a colorimetric endpoint that was read using ELISA reader. The optical density (OD) was measured by spectrophotometer (MULTISKAN EX, Thermo Electron Corporation) at 450 nm. A standard curve was prepared and the concentration of the CRP determined. Abnormal result of the inflammation marker was defined as that above the upper limit of ˃10mg/l.

HOMA-IR

The homeostasis model assessment index-insulin resistance (HOMA-IR), which is based on fasting sample insulin and glucose concentrations measured in a single blood sample, was used to calculate insulin resistance [2,3]. The HOMA-IR was determined from the equation: insulin resistance = [fasting insulin (μIU/ml) x fasting glucose (mmol/L)]/ 22.5. Abnormal HOMA-IR was defined as that above the upper limit ≥ 25.

Lipid Profile

Triglyceride, total cholesterol, HDL-cholesterol were assayed using Envoy^®^ 500 reagents (Vital Diagnostics, USA) according to the manufacture’s specification on BT 5000^®^ Random Access Chemistry Analyzer ( Biotecnica, Italy). The enzymatic hydrolysis of triglycerides produces glycerol in the sample, which is converted to glycerol phosphate in the presence of adenosine triphosphate (ATP) and glycerol kinase (GK). The resulting glycerol phosphate is oxidized in the presence of phosphate oxidase (GPO) to produce hydrogen peroxide (H_2_O_2_). The H_2_O_2_ reacts with p-chlorophenol and 4-aminoantipyrine (4-AAP) in the reagent to produce a red dye whose maximum absorption at 510 nm. HDL-cholesterol was separated from low density lipoprotein and very low density lipoprotein by selective precipitation with phosphotungsten acid in the presence of Mg^2+^ ions. HDL-cholesterol which remains in the supernatant was separated after centrifugation and its concentration was determined by the cholesterol oxidase method. LDL-cholesterol was calculated based on Friedwald’s equation; LDL-cholesterol = total cholesterol-(triglycerides/2.2 +HDL) mmol/L [4]

Chromium

Chromium was measured with an atomic Absorption Spectrometer (VARIAN AA 240FS- Atomic Absorption Spectrometer). 2.0 ml of blood were weighed and dispensed into a previously acid washed labelled 100ml polytetraflouroethylene (PTFE) Teflon bomb containing 6ml of concentrated nitric acid (65 % HNO_3_,) and 1ml of hydrogen peroxide (30 % H_2_O_2_). The mixture was digested in milestone microwave (labstation ETHOS 900, INSTR: MLS-1200 MEGA). After digestion, the Teflon bombs, mounted on the microwave carousel were cooled in a water bath to reduce internal pressure and allow volatilized material to re-stabilize and the digest aspirated into the atomic absorption spectrometer. The spectrometer was operated at 357.9 nm wavelength, 0.2 nm Slit, 7mA lamp current cathode lamp (Lumina, from Hollow Cathode Lamp) and fuel-rich (yellow) air-acetylene flame. Samples were read five times at a 1 second integration setting, as were appropriate standards. The absorbance values were used in the calculation of the concentrations of the metal. Reference standards: 1000 mg/L of chromium were prepared by dissolving 3.735 g of potassium chromate (K2CrO4), in deionized water and dilute to 1 liter with deionized water, blanks and duplicates of samples were digested in the same conditions as the samples. These served as internal controls (Reference standards used are from FLUKA ANALYTICAL, Sigma-Aldrich Chemie GmbH, Switzerland). 2% ammonium chloride (NH4Cl) was added to sample and standard controls solutions to reduce the interference caused by iron,whilst that of excess phosphate which depress the chromium response was overcome by the addition of calcium in the same order as was described for the ammonium chloride [5].

References

1. Chobanian AV, Bakris GL, Black HR, Cushman WC, Green LA, Izzo JL, Jr., Jones DW, Materson BJ, Oparil S, Wright JT, Jr., Roccella EJ: Seventh report of the Joint National Committee on Prevention, Detection, Evaluation, and Treatment of High Blood Pressure. *Hypertension* 2003; 42:1206-52.
2. Vogeser M, Parhofer KG: Comparison of automated insulin assays. *Clin Lab* 2007; 53:557-60.
3. Inaba M, Nishizawa Y, Mita K, Kumeda Y, Emoto M, Kawagishi T, Ishimura E, Nakatsuka K, Shioi A, Morii H: Poor glycemic control impairs the response of biochemical parameters of bone formation and resorption to exogenous 1,25-dihydroxyvitamin D3 in patients with type 2 diabetes. *Osteoporos Int* 1999; 9:525-31.
4. Friedewald WT, Levy Rl, Fredrickson DS. Estimation of the concentration of low density lipoprotein cholesterol in plasma, without use of the preparative ultracentrifuge.*Clin Chem*. 1972; 18:499–502.
5. Cookbook MAD: Milestone application notes for digestion. 1996.
